# Supplementary material for: Validation of the brief physical activity assessment tool: Comparison of telephone and in-person administration
Source: PLoS One. 2025 Jan 24;20(1):e0317614. doi: 10.1371/journal.pone.0317614 (PMC11760019; doi:10.1371/journal.pone.0317614)
Supplement: Attachment — Submitted filename: reviewer_letter.docx [file pone.0317614.s002.docx]

November 19^th^ 2024

Comments to the Author

4. Have the authors made all data underlying the findings in their manuscript fully available?

The PLOS Data policy requires authors to make all data underlying the findings described in their manuscript fully available without restriction, with rare exception (please refer to the Data Availability Statement in the manuscript PDF file). The data should be provided as part of the manuscript or its supporting information, or deposited to a public repository. For example, in addition to summary statistics, the data points behind means, medians and variance measures should be available. If there are restrictions on publicly sharing data—e.g. participant privacy or use of data from a third party—those must be specified.

Reviewer #1: Yes

Reviewer #2: Yes

Reviewer #3: (No Response)

Response: The data availability statement has been added during revision 1. Please find page x, lines xx: All data supporting the findings are available from the Faculty of Medicine of University of Porto for all interested researchers who meet the criteria for access to confidential data. Because these data include patients’ personal information, the Ethical Committees that approved the study does not recommend that such data be made public unnecessarily. Please contact Cristina Jácome, the corresponding author at cjacome@med.up.pt, or the Faculty of Medicine of University of Porto at fmup@med.up.pt to request the data.

6. Review Comments to the Author

Reviewer #3: I would first like to thank the authors for the revision of the manuscript and the point-by point explanations. One analysis that could enrich the manuscript would be to analyze according to sex only; so separately for both sexes, one row for females and one row for males. Their activity levels might be quite different and their expressions also might be different. Seeing the results of these would be interesting.

Response: Gender analyses were not included in the study for two primary reasons. First, addressing gender differences was not one of our main study objectives. Second, we believed that subdividing each age group by gender would yield insufficient statistical power to adequately address the questions posed, and analyzing gender differences without considering age range did not align with our hypothesis that age would influence questionnaire responses. We appreciate your understanding of our perspective.

Nevertheless, we acknowledge the significance of investigating gender differences in physical activity, and we are currently preparing a manuscript that analyzes these data based on the questionnaire.
